# Interrelationships between moral sensitivity, empathy, and perceived professional preparedness among undergraduate nursing students: a path analysis

**DOI:** 10.3389/fmed.2026.1824923

**Published:** 2026-06-03

**Authors:** Bayan Alilyyani, Mohammed Almalki, Fahad M. Althobaiti, Ibtihal Almalki, Lailani Sacgaca, Petelyne Pangket

**Affiliations:** 1 Department of Nursing Management and Education, College of Nursing, Taif University, Taif, Saudi Arabia; 2 Department of Community and Mental Health Nursing, College of Nursing, Taif University, Taif, Saudi Arabia; 3 Department of Maternity and Neonate Nursing, College of Nursing, Taif University, Taif, Saudi Arabia; 4 Department of Medical-Surgical Nursing, College of Nursing, Taif University, Taif, Saudi Arabia

**Keywords:** empathy, moral sensitivity, nursing education, professional preparedness, structural equation modeling, undergraduate nursing students

## Abstract

**Introduction:**

Empathy and moral sensitivity have been increasingly identified as important psychosocial resources that support the development of nursing students’ ethical awareness, relational practice, and professional identities. Strengthening nursing students’ capacity for empathic and morally sensitive practice within undergraduate education may also support their confidence in managing the complexity of clinical interactions and providing patient-centered care. This study aimed to examine an integrated structural model in which empathy and moral sensitivity were specified as exogenous constructs predicting undergraduate nursing students perceived professional preparedness for future nursing roles.

**Methods:**

This cross-sectional correlational study used path analysis based on partial least squares structural equation modeling (PLS-SEM) of data from 402 undergraduate nursing students enrolled in a Saudi Arabian nursing college. Participants completed validated instruments to assess empathy, moral sensitivity, and perceived professional preparedness for future nursing roles. Refined versions of all three instruments demonstrated high reliability and convergent validity.

**Results and Discussion:**

The path coefficients for the model were positive and significant, accounting for 70.3% of the variance in perceived professional preparedness. Both empathy (*β* = 0.594, *p* < 0.001) and moral sensitivity (*β* = 0.275, *p* < 0.001) positively predicted perceived professional preparedness.

## 1 Introduction

Moral sensitivity and empathy among nursing students are interrelated and contribute to the development of professional values that support ethical nursing practice and professional identity. In this study, and consistent with Rest’s Four-Component Model, moral sensitivity is defined as the capacity to recognize ethically relevant aspects of clinical situations and to anticipate how one’s behavior may affect others, providing a foundation for moral judgment, motivation, and character (1–3, 32). Empathy is conceptualized as a sociocognitive capacity to understand and share patients’ experiences and feelings and respond in a caring, patient-centered manner (4, 5). In the broader framework of emotional intelligence (EI), empathy and relationship management are central components that enable nursing students to recognize patients’ emotions and respond appropriately in interpersonal interactions (6–8). The development of strong professional values is related to greater moral sensitivity and empathic tendencies and is fundamental to both identity formation and ethical practice (9, 10, 33). A strong sense of moral sensitivity allows students to see how their behavior affects others, make ethically informed decisions, and apply professional and ethical standards in practice (34). Empathy contributes to students’ ability to provide patient-centered care, foster relationships with patients based on caring and justice, and deliver genuine care (11, 35). From a developmental perspective, Hoffman’s theory defines empathy as a socio-cognitive capacity that develops from basic affective sharing to principled concern for others’ welfare through cognitive growth and socialization (12, 36). Together, these two capacities can be viewed as complementary moral and socio-cognitive resources that develop over time and form the basis for caring behavior in clinical settings.

Despite the growing evidence for the importance of moral sensitivity and empathy, a gap remains between the values expected of future nurses and those demonstrated in practice (10, 33). Models of relational care, including Travelbee’s human-to-human relationship model, emphasize authentic interpersonal relationships and reciprocal understanding, providing a foundation for developing professional values and empathetic nursing practices (35, 37). Professional development is a long-term process that depends on supportive learning environments in both classroom and clinical settings and ongoing guidance from educators, and strengthening these professional and ethical values in nursing students is likely to improve care quality by enhancing their sense of preparedness to assume professional roles and manage ethically complex situations in the future (38, 39).

Although moral sensitivity and empathy have been widely studied, relatively few studies have examined how these two constructs relate to nursing students’ perceptions of professional preparedness. Most existing research has focused on these concepts separately as components of professional values rather than integrating them with perceived preparedness in a single model (9, 10). This gap underscores the need to clarify whether and how moral sensitivity and empathy influence students’ perceptions of professional preparedness and guide educational strategies for enhancing ethical and professional development. This study was guided by Rest’s perspective, which views moral sensitivity as a developing capacity to recognize ethically relevant features of situations, and Hoffman’s perspective, which conceptualizes empathy as a developing sociocognitive capacity that progresses from affective sharing to principled concern for others (32, 36).

In undergraduate nursing education, higher levels of moral sensitivity and empathy are expected to enhance ethical awareness, relational competence, and confidence in managing complex clinical situations, thereby contributing to the perceived preparedness for professional roles. Accordingly, a structural model was specified in which moral sensitivity and empathy were exogenous constructs, and perceived professional preparedness was the endogenous construct. In line with this model, two hypotheses were proposed: (H1) higher moral sensitivity is positively associated with greater perceived professional preparedness, and (H2) higher empathy is positively associated with greater perceived professional preparedness among undergraduate nursing students.

## 2 Materials and methods

### 2.1 Study design

This cross-sectional, correlational study used path analysis to test an integrated structural model of the associations among moral sensitivity, empathy, and perceived professional preparedness within a theoretically informed framework.

### 2.2 Population and sampling

The study population comprised all undergraduate nursing students enrolled in the College of Nursing at Taif University during the first semester of the 2025-2026 academic year. This included male and female students across different year levels in the bachelor’s nursing program, while excluding students enrolled in master’s and bridging programs and those who provided incomplete responses to the questionnaires. A non-probability convenience sampling technique was used, whereby eligible undergraduate students who were willing to participate were invited, resulting in 420 survey responses. After reviewing the data for completeness, 18 incomplete submissions were excluded, leaving a final analytical sample of 402 participants.

Although an *a priori* G*Power calculation for a multiple regression model with two main predictors (moral sensitivity and empathy), a medium effect size (*f*^2^ = 0.15), *α* = 0.05, and power = 0.80 indicated a minimum required sample of approximately 150 participants, additional criteria related to the planned path analysis were considered. Given the number of indicators for perceived professional preparedness and the overall complexity of the hypothesized model, a sample size above 300 was deemed advisable to obtain stable and precise parameter estimates. Therefore, the sample size of 402 students satisfied the requirements.

#### 2.2.1 Instruments

The questionnaire consisted of four sections. The first section collected sociodemographic data, including gender, age, marital status, place of residence, GPA, year level, and primary clinical rotation area (e.g., medical–surgical, critical care, maternal–child, or community health).

The second section was the Moral Sensitivity Questionnaire (31), which includes 11 items (two reverse-scores) rated on a 6-point Likert scale from 1 (completely disagree) to 6 (completely agree), yielding total scores ranging from 11 to 66, with higher scores indicating higher moral sensitivity. Prior studies have reported acceptable content validity (CVI ≈ 0.78) and Cronbach’s alpha values of approximately 0.75-0.76. This instrument captures key aspects of moral sensitivity as defined in this study, including the recognition of ethically salient situations in clinical practice, awareness of value conflicts, and a sense of responsibility toward patients and colleagues.

The third section included the Nurse Empathy Scale (13), a 16-item instrument rated on a 5-point Likert scale from 1 (strongly disagree) to 5 (strongly agree), with total scores ranging from 16 to 80, with higher scores indicating greater empathy. It demonstrated strong internal consistency, with a Cronbach’s alpha of approximately 0.89. Consistent with our conceptualization of empathy, this scale assesses both cognitive and affective components, such as perspective-taking, understanding patients’ feelings and experiences, and responding in a supportive, patient-centered manner.

The fourth section was the Perceived Professional Preparedness of Senior Nursing Students Questionnaire (14), consisting of 20 items rated on a 5-point Likert scale from 1 (completely disagree) to 5 (strongly agree), with raw scores transformed to a 0–100 scale, where scores below 25% indicated weak, 25–50% moderate, 50–75% good, and above 75% excellent perceived professional preparedness. This instrument has shown acceptable content validity (CVI ≈ 0.72) and reliability (Cronbach’s alpha ≈ 0.75) in prior studies. In line with our definition of professional preparedness, this questionnaire evaluates nursing students’ perceived readiness to assume their future roles, including confidence in clinical decision-making, communication, and delivering ethical, person-centered care.

In this study, the internal consistency and convergent validity of these three latent constructs (empathy, moral sensitivity, and perceived professional preparedness) were evaluated. Table 1 presents the Cronbach’s alpha, composite reliability, and average variance extracted (AVE) values, all of which indicate the satisfactory reliability and convergent validity of the measurement model.

**Table 1:** Measurement model: reliability and convergent validity.

Construct	Cronbach’s *α*	Composite reliability (rho_a)	Composite reliability (rho_c)	AVE
Empathy	0.941	0.942	0.948	0.548
Moral sensitivity	0.916	0.918	0.931	0.600
Perceived professional preparedness	0.969	0.969	0.971	0.641

AVE, average variance extracted.

During the preliminary measurement-model assessment, three items with standardized factor loadings below 0.40 were removed (one item from the Moral Sensitivity Questionnaire and two items from the Perceived Professional Preparedness Questionnaire), and subsequent analyses were conducted using this refined version of the instrument. In this sample, these refined scales demonstrated excellent internal consistency, with Cronbach’s alpha values exceeding 0.90 and AVE values above 0.50, which were substantially higher than those in the validation studies. Because the removal of items alters the original scale configuration, the resulting versions should be regarded as modified instruments that require further psychometric evaluation, particularly of content validity, before being considered directly comparable to the original questionnaire. The assumption that the refined scales retained their intended content domains is based on the remaining items and theoretical rationale; however, a formal reassessment of content validity was beyond the scope of this study.

### 2.3 Data collection

Data was collected using a self-administered online survey constructed using Google Forms during the second semester in October 2025. After obtaining approval from the Institutional Review Board of Taif University, approval was also obtained from the Dean of the College of Nursing to approach eligible students during regular classes.

At the beginning of each selected course, the researchers held a short introduction session that outlined the study goals, how it would be carried out, the expected duration for its completion, the possible risks and benefits associated with participation, and the fact that participation was completely voluntary. Interested students were given a QR code that allowed them to access the web-based survey by scanning it and completing the questionnaire on their devices at their convenience. The first screen of the online survey contained an information sheet and an informed consent form. Upon agreeing to participate, electronic consent was obtained, and the participants were immediately taken to the remainder of the survey. No identifiable data (i.e., names or ID numbers) were recorded; therefore, all questionnaires were reviewed before analysis, and those that did not contain complete or consistent data were removed from the database used for analyses.

### 2.4 Statistical analysis

Statistical analyses were performed using IBM SPSS Statistics 26.0 and AMOS 26.0 (IBM Corporation, Armonk NY, United States). IBM SPSS Statistics 26.0 was used for descriptive statistics and preliminary data screening. AMOS 26.0 was used for the graphical visualization of the structural model. Primary structural analyses were conducted using partial least squares structural equation modeling (PLS-SEM) procedures. Partial Least Squares Structural Equation Modeling (PLS-SEM) was conducted using SmartPLS version 4.0. Descriptive statistics (mean ± standard deviation, frequency, and percentage) were calculated to describe the participants’ sociodemographic characteristics and study variables. The psychometric properties of the constructs investigated in this study (internal consistency: Cronbach’s alpha; composite reliability; and convergent validity: average variance extracted [AVE]) were tested.

The model fit was evaluated using the SRMR, d_ULS, d_G, and NFI as common approximate fit indicators for the variance-based PLS-SEM. These indicators estimate the degree to which a model approximates the observed covariance structures. SRMR was treated as the primary approximate fit indicator, with values < 0.08 interpreted as indicating acceptable fit. The d_ULS and d_G values were reported as supplementary discrepancy measures; however, because bootstrap-based HI95/HI99 reference thresholds were not computed in this analysis, these indices were not used as definitive fit criteria. NFI values >0.80 are often interpreted as acceptable, and NFI values >0.90 as an indication of a good fit, especially in a more confirmatory approach. However, because the global model fit in PLS-SEM is inherently less comprehensive than covariance-based SEM, all fit indicators were considered secondary to the examination of the structural path estimates and their explained variances (*R*^2^).

Given the cross-sectional design, the structural paths were specified to represent hypothesized relationships consistent with the theoretical model, and these findings reflect associations rather than definitive causal effects. Partial least squares structural equation modeling (PLS-SEM) with path analysis was used because the primary aim was to estimate the associations and explain variance in perceived professional preparedness within a moderately complex structural model, emphasizing prediction rather than strict covariance-based model confirmation. Standardized path coefficients (*β*), standard errors, *t*-values, *p*-values, and *R*^2^ for the endogenous constructs were reported, with statistical significance set at *p* < 0.05.

### 2.5 Ethical considerations

This study was conducted in accordance with the national and institutional ethical standards for research ethics. The Scientific Research Ethics Committee of Taif University (No: 47-034, Date: 05/10/2025) approved this study prior to data collection. All participants provided electronic informed consent via the first page of the online survey, which included an information sheet describing the study’s purpose, procedures, potential risks and benefits, and the voluntary nature of participation.

Participants’ privacy was assured by assigning them coded numbers instead of identifying details in the dataset. Participants were advised that they could withdraw at any time and/or refuse to participate without any penalties.

## 3 Results

Table 1 details the psychometric performance of the three latent constructs. All demonstrated strong psychometric performance, with Cronbach’s alpha values above 0.90 and composite reliabilities (*ρ_a_* and *ρ_c_*) exceeding 0.91, indicating excellent internal consistency across the measurement model. The AVE values for empathy (0.548), moral sensitivity (0.600), and perceived professional preparedness (0.641) were all greater than 0.50, confirming adequate convergent validity for subsequent structural analyses. Notably, the present study yielded substantially higher internal consistency coefficients for all three scales compared with the original validation studies, in which Cronbach’s alpha values for the Moral Sensitivity Questionnaire and the Perceived Professional Preparedness Questionnaire were approximately 0.75-0.76. These improvements indicate that the remaining items functioned coherently in this sample and yielded stronger reliability and convergent validity. However, because the item deletions changed the original content structure, these refined versions should be interpreted as modified forms of the Moral Sensitivity Questionnaire and the Perceived Professional Preparedness Questionnaire, and scores cannot be assumed to be directly comparable with those from studies using the full original scale.

The sample comprised 402 nursing students, most of whom were female (63.2%), with males representing 36.8% of the participants. The majority were young adults, with 26.9% aged 18–20 years, 30.8% aged 21–25 years, and 32.3% aged 26–30 years, whereas only 10.0% were 31 years or older.

Almost all participants were single (94.8%), and only 5.2% were married. Regarding academic level, 2.0% were in the first year, 37.8% in the second year, 44.3% in the third year, and 15.9% in the fourth year of the nursing program.

Clinical experience was distributed across multiple settings. The most commonly reported areas were “others” (14.4%), recovery/operating room (13.7%), primary health care clinics (12.9%), and surgical wards (11.9%), whereas fewer students had experience in psychiatric wards (10.9%), outpatient departments (9.7%), emergency departments (6.7%), medical wards (6.2%), maternity and gynecology wards (5.2%), pediatric wards (4.2%), and intensive or critical care units (ICU/CICU/SICU/CCU) (4.0%) (see Table 2).

**Table 2:** Socio-demographic profile of nursing students (*n* = 402).

Variable	Category	*n*	%
Gender	Female	254	63.2
	Male	148	36.8
Age (years)	18–20	108	26.9
	21–25	124	30.8
	26–30	130	32.3
	≥31	40	10.0
Marital status	Single	381	94.8
	Married	21	5.2
Year level in nursing education	1st year	8	2.0
	2nd year	152	37.8
	3rd year	178	44.3
	4th year	64	15.9
Area of clinical experience	Others	58	14.4
	Recovery/operating room	55	13.7
	Primary health care clinics	52	12.9
	Surgical ward (male/female)	48	11.9
	Psychiatric ward (male/female)	44	10.9
	Outpatient department	39	9.7
	Emergency department	27	6.7
	Medical ward (male/female)	25	6.2
	Maternity and gynecology ward	21	5.2
	Pediatric ward	17	4.2
	ICU/CICU/SICU/CCU	16	4.0

Table 3 shows that the PLS-SEM model demonstrated acceptable approximate fit, with SRMR values of 0.048 for both the saturated and estimated models, which are below the commonly recommended threshold of 0.08. The additional indices (d_ULS, d_G, and NFI = 0.814) were interpreted cautiously as supplementary indicators supporting approximate model adequacy rather than definitive evidence of global fit.

**Table 3:** Model fit indices for the PLS-SEM model.

Fit index	Saturated model	Estimated model
SRMR	0.048	0.048
d_ULS	2.218	2.218
d_G	1.287	1.287
NFI	0.814	0.814

SRMR, standardized root mean square residual; NFI, normed fit index.

Table 4 shows that the model explained a substantial proportion of the variance in perceived professional preparedness, with an *R*^2^ value of 0.703 and an adjusted *R*^2^ of 0.702.

**Table 4:** Explained variance in perceived professional preparedness.

Endogenous variable	*R* ^2^	Adjusted *R*^2^
Perceived professional preparedness	0.703	0.702

Table 5 indicates that empathy and moral sensitivity had statistically significant positive relationships with nursing students’ perceived professional preparedness within the structural model. The association between empathy and perceived professional preparedness was strong and positive (*β* = 0.594, SE = 0.095, *t* = 6.233, *p* < 0.001), whereas the association between moral sensitivity and perceived professional preparedness was more modest but still positive and statistically significant (*β* = 0.275, SE = 0.078, *t* = 3.519, *p* < 0.001). This suggests that higher levels of empathy and moral sensitivity are associated with greater perceived professional preparedness among nursing students.

**Table 5:** Structural path coefficients (standardized associations) from empathy and moral sensitivity to perceived professional preparedness.

Path	*β*	STDEV	*t*-value	*p*-value
Empathy → perceived professional preparedness	0.594	0.095	6.233	<0.001
Moral sensitivity → perceived professional preparedness	0.275	0.078	3.519	<0.001

Path coefficients are estimated associations within a cross-sectional structural model and should not be interpreted as evidence of definitive causal effects.

**Figure 1:**
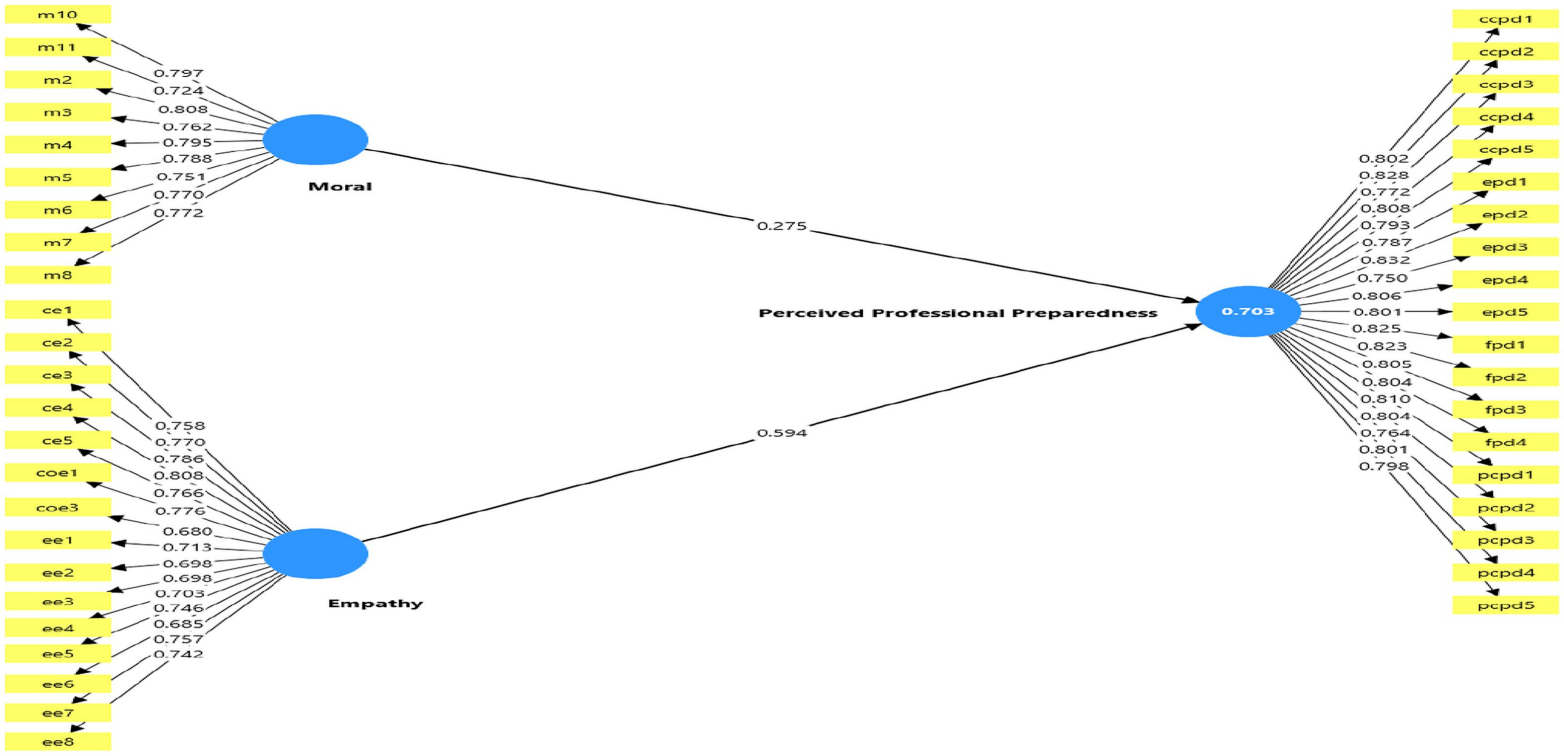
Structural path model showing the association of empathy and moral sensitivity on perceived professional preparedness among undergraduate nursing students.

Figure 1 provides a visual representation of the structural path model, depicting the standardized paths from empathy and moral sensitivity to perceived professional preparedness and the factor loadings for each observed indicator.

Table 6 presents the results of the sensitivity analysis by year. Among the second-year students (*n* = 152), empathy (*β* = 0.425, *p* < 0.001) and moral sensitivity (*β* = 0.398, *p* < 0.001) contributed nearly equally to perceived professional preparedness (*R*^2^ = 0.621). Among third-year students (*n* = 178), empathy (*β* = 0.640, *p* < 0.001) emerged as the stronger predictor, while moral sensitivity (*β* = 0.241, *p* = 0.001) remained significant (*R*^2^ = 0.725). Among fourth-year students (*n* = 64), empathy was the sole significant predictor (*β* = 0.876, *p* < 0.001), whereas moral sensitivity was not statistically significant (*β* = 0.088, *p* = 0.171), and the model explained 88.9% of the variance (*R*^2^ = 0.889). The first-year subgroup (*n* = 8) was excluded because of insufficient sample size. Across year levels, the direction of the associations remained consistent with the main model; however, empathy became the dominant predictor as students advanced, while moral sensitivity lost significance by the final year.

**Table 6:** Sensitivity analyses stratified by gender and year level examining predictors of perceived professional preparedness.

Subgroup	*n*	Predictor	*β*	*p*-value	*R* ^2^	Interpretation
Gender						
Female	254	Empathy	0.544	<0.001	0.636	Significant positive association
		Moral sensitivity	0.294	<0.001	0.636	Significant positive association
Male	148	Empathy	0.635	<0.001	0.777	Significant positive association
		Moral sensitivity	0.286	<0.001	0.777	Significant positive association
Year level						
2nd year	152	Empathy	0.425	<0.001	0.621	Significant positive association
		Moral sensitivity	0.398	<0.001	0.621	Significant positive association
3rd year	178	Empathy	0.640	<0.001	0.725	Significant positive association
		Moral sensitivity	0.241	0.001	0.725	Significant positive association
4th year	64	Empathy	0.876	<0.001	0.889	Significant positive association
		Moral sensitivity	0.088	0.171	0.889	Not statistically significant
1st year	8	—	—	—	—	Sample size too small for stable analysis

*β*, standardized regression coefficient; *R*^2^, explained variance in perceived professional preparedness; Separate subgroup regression analyses were conducted for each subgroup. The first-year subgroup was excluded from interpretation because of the very small sample size.

## 4 Discussion

The findings of this study indicate that both empathy and moral sensitivity are significantly associated with undergraduate nursing students’ perceived professional preparedness, with empathy demonstrating a stronger association than moral sensitivity. Because these findings concern perceived rather than observed preparedness, future studies should triangulate self-report scales with peer and faculty ratings, behavioral observations in simulated or real clinical encounters, and patient-reported experiences to better approximate actual professional readiness in the field.

The results of this study suggest that when students report high levels of empathy, they are more confident in managing clinical encounters, communicating with patients and families, and responding appropriately to patients’ needs. Moral sensitivity also adds explanatory power, indicating that recognizing ethically salient aspects of clinical situations helps students anticipate and manage moral complexity in their future roles.

The substantial variance explained in perceived professional preparedness, together with the strong direct paths from empathy and moral sensitivity, underscores the critical role that these psychosocial resources play in how prepared students feel to enter the profession. Rather than being peripheral traits, empathy and moral sensitivity appear to be central to students’ professional identity formation and to their sense of competence in applying knowledge, exercising judgment, and delivering ethically grounded, person-centered care.

### 4.1 Professional preparedness and the role of empathy

Empathy showed a strong association with nursing students’ perceived professional preparedness. This association was stronger than that observed for moral sensitivity, indicating that in this sample, empathy scores were more closely linked to perceived readiness for the first year of practice than to ethical awareness of clinical situations. These findings suggest that students’ feelings about their professional capabilities are most strongly tied to their perceived capacity to build and maintain therapeutic relationships with patients and families and interact effectively during emotionally demanding encounters. Moral sensitivity was also significantly associated with perceived professional preparedness, indicating that students recognize the importance of ethics in care, although ethical awareness does not appear to be the primary factor shaping their sense of readiness for day-to-day clinical demands at this stage. However, the relatively high explained variance for perceived professional preparedness (*R*^2^ = 0.703) should be interpreted with caution, as some of these variances may be attributable to shared method variance arising from the single-source, self-report design rather than substantive relationships among the constructs.

The current results align with a substantial body of literature demonstrating that empathy is related to person-centered care, therapeutic communication, reduced stress in clinical practice, and the development of overall competence among nursing students (15–19). Prior research has also shown that higher empathy among nursing and medical students is associated with improved patient outcomes, reduced patient distress, and patients’ perceptions of higher-quality care (16, 20). Simultaneously, previous studies have highlighted the influence of additional factors such as emotional intelligence, self-compassion, resilience, and the quality of both curriculum and clinical experiences within the broader network of determinants of professional preparedness (7, 21, 40).

The finding that empathy was a stronger predictor of students’ perceived preparedness in this model suggests that educational strategies should prioritize opportunities that actively develop empathic abilities, rather than relying solely on didactic ethics content. From an EI perspective, these results indicate that emotionally relevant capacities such as empathy, emotion recognition, and relationship management are central to how students appraise their readiness for practice (6–8). Integrating EI-focused learning activities, including training in empathic communication and management of emotionally demanding encounters, may therefore be a practical strategy for enhancing nursing students perceived professional preparedness.

### 4.2 Moral sensitivity as a supporting resource for professional preparedness

Students who are sensitive to ethical issues in health care have been found to be marginally associated with their perceptions of professional readiness. These findings suggest that the ability to identify the ethical components of patient care can contribute to, but does not by itself ensure, a sense of professional readiness. Developing an awareness of one’s responsibility to act professionally (moral sensitivity) appears to provide a foundation for the development of other competencies, such as empathy and clinical knowledge. Prior research supports this analysis, with studies showing that higher levels of moral sensitivity among nursing students and practicing nurses are associated with more professional and caring behaviors and an intuitive awareness of moral dilemmas and ethical responsibilities, which can improve how individuals respond to such situations and support their readiness to practice (11, 22–24). However, moral sensitivity is only one of several psychological resources, including self-efficacy, that contribute to person-centered care and overall quality of care (25, 26). When high moral sensitivity is not supported by sufficient professional autonomy or institutional support, students may recognize ethical issues but feel unable to act on them, reducing confidence and limiting the extent to which moral sensitivity contributes to their preparedness (27). Thus, nursing education programs may need to create learning environments that help students translate the recognition of ethical concerns into confidence in their ability to act appropriately in the future.

### 4.3 Explanatory power of the integrated model

The integrated model explained 70.3% of the variance in perceived professional preparedness, suggesting that this outcome reflects the combined influence of moral sensitivity and empathy rather than the effect of either resource alone. However, this high level of explained variance may partly reflect the characteristics of this single institution and cohort, including a supportive educational climate and specific curricular emphasis. Consequently, the strength of these associations and the overall model performance are likely to apply to nursing programs with similar cultural, institutional, and curricular profiles. Within this framework, empathy provides the socio-cognitive tools needed to enact care, while moral sensitivity anchors these responses in professional responsibilities, together facilitating professional identity formation and bridging the gap between recognizing ethical issues and enacting relational responses.

These findings are consistent with empirical evidence indicating that both moral sensitivity and empathy contribute to professional values and competence (10, 28). Prior research has shown that empathy can serve as a mechanism through which moral awareness is expressed in practice and may mediate the relationship between moral sensitivity and professional competence (9, 10, 28). However, some studies have reported much weaker relationships, with models explaining only a small proportion of variance or showing diminished associations due to cultural factors, curriculum design, or phase of training (29, 30). Accordingly, these results should be regarded as hypothesis-generating for similar Saudi and Gulf undergraduate nursing programs rather than definitive evidence that the observed patterns and magnitudes of effects will generalize to other educational systems in the future (7).

Given that the integrated model accounted for a large proportion of variance in professional preparedness, educational programs should prioritize curricula that foster the concurrent development of both moral sensitivity and empathy. Institutions should also consider cultural context and training stage by periodically evaluating the model across cohorts to monitor its effectiveness and adjust pedagogical approaches as needed.

### 4.4 Implications for nursing education

Empathy and sensitivity to ethical issues (moral sensitivity) should be regarded as central competency areas in nursing programs and articulated as explicit learning outcomes or competency statements, rather than being treated as merely idealistic or desirable personal qualities. The rationale for this position is that professionalism includes the capacity to form therapeutic relationships with patients, and students’ ability to respond to patients’ emotional needs is directly linked to their readiness for entry-level nursing practice. In addition to enhancing readiness for practice, moral sensitivity strengthens students’ preparedness to address the complexities of ethical decision-making in clinical settings.

The significant amount of variance in how prepared nursing students felt to provide patient care that was explained by empathy and moral sensitivity is a strong reason for educators to focus on developing both abilities. Education programs for nursing students in similar institutional and cultural contexts could use experiential, or “hands-on,” methods to support traditional didactic ethics teaching. Developing learning activities that explicitly relate classroom content to clinical experience will help to support nursing students’ professional development and strengthen their confidence when making ethically based judgments in patient care.

### 4.5 Implications for quality assurance and program evaluation

The strong scale reliabilities in this study suggest that the adapted versions could serve as useful tools for assessing curricular effectiveness in similar educational environments. These measures can be used by academic administrators to assess cohort progress in empathy, moral sensitivity, and perceived professional preparedness at graduation, and to evaluate the effects of new teaching methods or curriculum changes. Until further studies are completed, however, it would be premature to conclude that the modified scales are fully valid; instead, the present findings should be interpreted as evidence of associations among the variables as measured by the adapted versions in this specific context. Therefore, the use of these adapted scales should be limited to institutions with comparable student demographics and program structures, and additional psychometric studies using a range of analytical techniques are needed before they are applied more broadly in other educational settings.

### 4.6 Implications for future research

Further studies are needed to see whether similar teaching approaches for improving empathy and moral sensitivity work well in other types of nursing programs and cultures. Studies that follow students over time or test specific teaching interventions could show whether these approaches lead to lasting improvements in these abilities and help new nurses feel better prepared and act ethically in their early clinical work.

### 4.7 Study limitations

The sample for this study was drawn from a single Saudi public university using a non-probability (convenience) sampling strategy, which restricts the generalizability of the results to nursing students in other programs, regions, and cultural settings. Although students from all four years of study were included, no longitudinal designs were used that would allow for comparisons of students’ empathy and moral sensitivity as they progressed through their education. The three constructs examined in this study, students’ empathy, moral sensitivity, and perceptions of their professional preparedness, were measured using self-administered Likert-type scales in a single survey session, which may have led to common method variance and inflated associations among the variables, thereby increasing the proportion of variance in preparedness explained by students’ self-ratings rather than observed performance. Accordingly, the relatively high explained variance for perceived professional preparedness ( $R^{2}=0.703$ ) should be interpreted with caution, as part of this value may reflect shared method variance rather than substantive relationships among the constructs.

The study did not incorporate objective indicators (such as OSCE scores, preceptor assessments, or patient-reported outcomes), which limits conclusions about how these psychosocial traits manifest in actual clinical practice. Although the modifications applied to the scales improved internal reliability, they also altered the original content domains, meaning that the revised instruments were not equivalent to the originals. Consequently, comparisons of mean scores or effect sizes with those of studies using the original scales should be interpreted cautiously. Subsequent studies should employ confirmatory factor analysis and evaluate the content validity of modified scales in diverse nursing student populations.

Furthermore, the structural model focused only on empathy, moral sensitivity, and perceived preparedness, omitting other likely determinants of readiness for practice, such as teaching quality, clinical workload, resilience, and features of the learning environment. The model therefore offers a partial account of the factors shaping student preparedness and underscores the need for longitudinal, multi-site research that incorporates a broader set of variables and tests the stability of the model and potential causal pathways over time.

## 5 Conclusion

The structural model indicated a statistically significant relationship between perceived professional preparedness and empathy. There were strong positive relationships between both moral sensitivity and empathy and students’ perceived preparedness; students who reported being more empathetic also expressed higher confidence in managing clinical encounters, communicating with patients’ family members, and attending to patients’ needs. Moral sensitivity also showed a statistically significant association with preparedness, implying that the capacity to recognize morally relevant issues is positively related to awareness of ethical complexity and adherence to professional ethics and conduct. These two constructs appeared to account for much of the variation in perceived preparedness and are therefore major components of professional identity formation.

Curriculum elements, including ethics training, structured reflection on clinical experiences, and experiential approaches such as simulation and relationship-centered learning, may be associated with higher levels of self-perceived preparation among undergraduate nursing students. Because the data for this study were collected at a single point in time (cross-sectional), it was not possible to draw causal conclusions about these relationships. Future longitudinal studies and cross-cultural comparisons are needed to examine whether these findings can be replicated in other nursing curricula and to clarify whether growth in empathy and moral awareness contributes to a smoother transition into first professional nursing roles.

## Data Availability

The raw data supporting the conclusions of this article will be made available by the authors, without undue reservation.
